# Rapid identification of a narcotic plant *Papaver bracteatum* using flow cytometry

**DOI:** 10.1007/s11418-014-0850-z

**Published:** 2014-06-22

**Authors:** Masako Aragane, Daisuke Watanabe, Jun’ichi Nakajima, Masao Yoshida, Masao Yoshizawa, Tomohiro Abe, Rei Nishiyama, Jin Suzuki, Takako Moriyasu, Dai Nakae, Hiroshi Sudo, Hiroyuki Sato, Atuyuki Hishida, Nobuo Kawahara, So Makabe, Ikuo Nakamura, Masahiro Mii

**Affiliations:** 1 Medicinal Plant Garden, Tokyo Metropolitan Institute of Public Health, 21-1 Nakajima’cho, Kodaira, Tokyo 187-0033 Japan; 2 Department of Pharmaceutical and Environmental Sciences, Tokyo Metropolitan Institute of Public Health, 3-24-1 Hyakunin’cho, Shin’juku, Tokyo, 169-0073 Japan; 3 Health and Safety Section, Bureau of Social Welfare and Public Health, Tokyo Metropolitan Government, 2-8-1 Nishi-Shin’juku, Shin’juku, Tokyo, 163-8001 Japan; 4 Faculty of Pharmaceutical Sciences, Hoshi University, 2-4-41 Ebara, Shinagawa, Tokyo 142-8501 Japan; 5 Okinawa Churashima Research Center, 888 Ishikawa, Motobu’cho Kunigami, Okinawa, 905-0206 Japan; 6 Hokkaido Division, Research Center for Medicinal Plant Resources, National Institute of Biomedical Innovation, 108-4 Ohashi, Nayoro, Hokkaido 096-0065 Japan; 7 Tsukuba Division, Research Center for Medicinal Plant Resources, National Institute of Biomedical Innovation, 1-2 Hachiman-dai, Tsukuba, Ibaraki 305-0843 Japan; 8 Laboratory of Plant Cell Technology, Graduate School of Horticulture, Chiba University, 648 Matsudo, Matsudo, Chiba 271-8510 Japan

**Keywords:** *Papaver bracteatum*, *Oxytona* section, DNA content, Flow cytometry, Identification of species, Morphological characteristics, PS-ID sequence, Thebaine

## Abstract

In May 2011, numerous poppy plants closely resembling *Papaver bracteatum* Lindl., a type of narcotic plant that is illegal in Japan, were distributed directly from several large flower shops or through online shopping throughout Japan, including the Tokyo Metropolitan area. In order to better identify the narcotic plants, the relative nuclear DNA content at the vegetative stage was measured by flow cytometric (FCM) analysis in 3 closely-related species of the genus *Papaver* section *Oxytona*, namely *P. orientale*, *P. pseudo*-*orientale,* and *P. bracteatum*, based on the difference between the chromosome numbers of these species. The results showed that the nuclear DNA content differed between these 3 species, and that most of the commercially distributed plants examined in this study could be identified as *P. bracteatum*. The remaining plants were *P. pseudo*-*orientale*, a non-narcotic plant. In addition, the FCM results for the identification of *P. bracteatum* completely agreed with the results obtained by the morphological analysis, the inter-genic spacer sequence of *rpl16*–*rpl14* (PS-ID sequence) of chloroplast DNA, and the presence of thebaine. These results clearly indicate the usefulness of FCM analysis for the identification of *P. bracteatum* plants, including when they are in their vegetative stage.

## Introduction

*Papaver bracteatum* Lindl. is cultivated as an ornamental plant in many countries. In Japan, however, it has been illegal to cultivate or even possess the plants of this species since June 19, 1990, because it contains a narcotic substance, thebaine [1].

*P. bracteatum* belongs to the genus *Papaver* section *Oxytona*, which consists of 3 species, *P. bracteatum* (2n = 2x = 14), *P. orientale* L. (2n = 4x = 28), and *P. pseudo*-*orientale* (2n = 6x = 42) [2, 3]. *P. orientale* and *P. pseudo*-*orientale* are allowed to be cultivated in Japan. Since all 3 species of the genus *Papaver* section *Oxytona* closely resemble each other in their morphology, especially at the young stages with only rosette leaves, there can be occasional problems with misidentifying the narcotic plant. It was thus necessary to develop a method to clearly discriminate *P. bracteatum* from the other 2 species.

In 1993, 3 years after the above-mentioned regulation was instituted [1], Kubota et al. [4] tried to identify the section *Oxytona* plants cultivated for ornamental purposes in Japan, and found that *P. bracteatum* was being cultivated in home gardens. In May 2003, potted plants with or without flowers closely resembling those of *P. bracteatum* were widely distributed commercially from large flower shops and through online shopping in Japan under the name of ‘oriental poppy’. Some of these plants were actually confirmed to be *P. bracteatum* by their morphological characteristics at the flowering stage, but the identification was very difficult in many samples that were at the vegetative stage. Because of such problems identifying the narcotic plants, it is important to establish an easy and quick method of identifying *P. bracteatum* even at the vegetative stage.

In this context, the present study was conducted to explore the usefulness of flow cytometric (FCM) analysis for the rapid discrimination of the 3 species of the genus *Papaver* section *Oxytona*, based on their differences in ploidy level. The FCM findings, in addition to the results of the analyses of morphological characteristics, chloroplast DNA sequences, and detection of thebaine and isothebaine, definitively distinguish the narcotic from the non-narcotic species.

## Materials and methods

### Plant materials

**Fig. 1:**
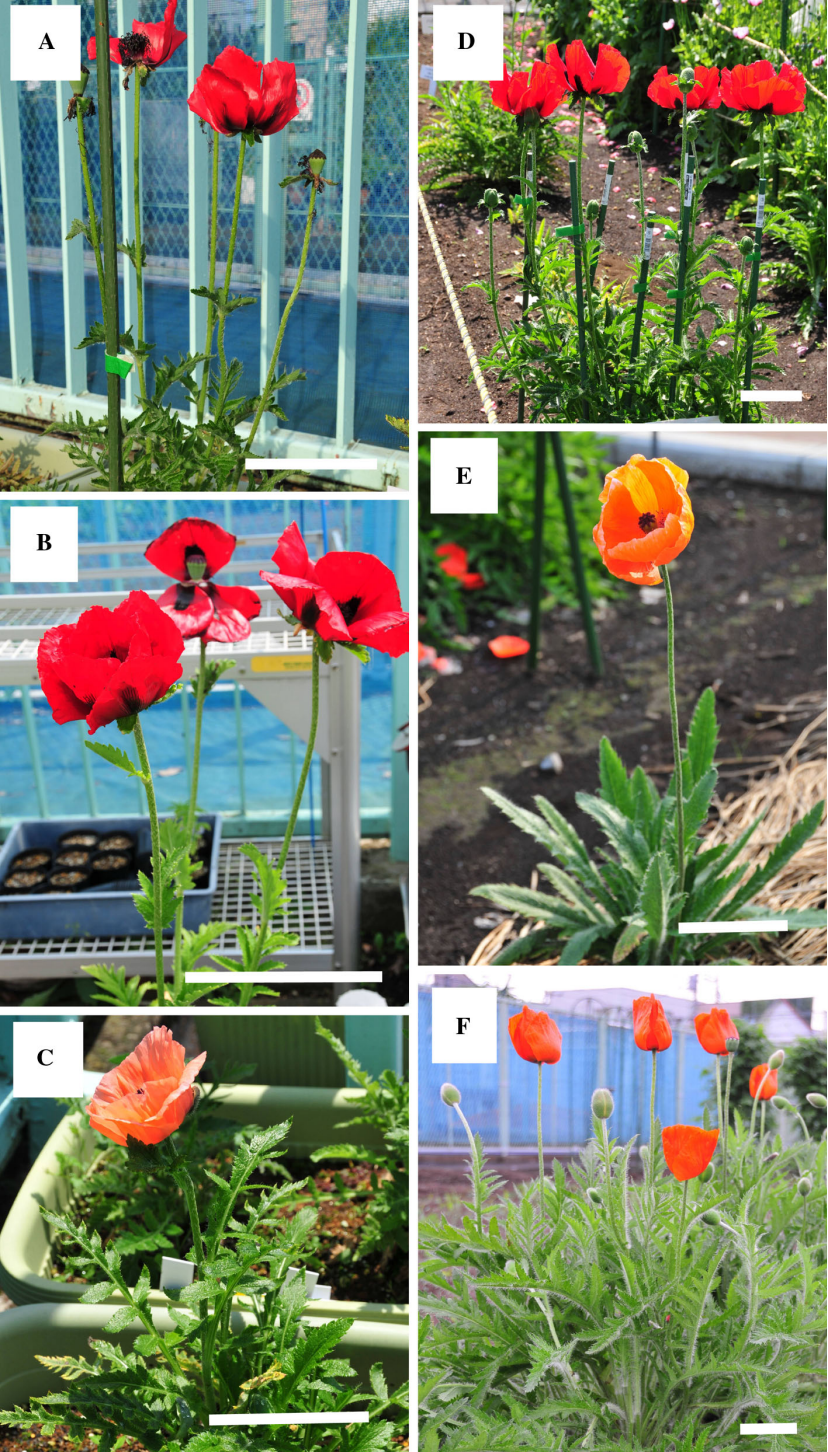
Plant materials. **a** Pa-1; **b** Pa-5; **c** Pa-12; **d** Pa-13; **e** Pa-14; **f** Pa-15. *Bar* 10 cm

A total of 12 plants, sold with the cultivar name of “oriental poppy” (*P. orientale*), were purchased in the Tokyo Metropolitan area and used as the test materials (Table 1; Fig. 1). Plant numbers Pa-1 to -10 were sold under the cultivar name of ‘Beauty of Livermere’, while Pa-11 and -12 were sold under the cultivar names of ‘Royal Wedding’ and ‘Prinzessin Victoria Louise’, respectively. At the initiation of this study (May 2011), these plants were mostly at the vegetative stages with only rosette leaves, but Pa-1, -2, and -7 had already attained the flowering stage (Table 1; Fig. 1). All of these plant materials were cultivated at the Medicinal Plant Garden, Tokyo Metropolitan Institute of Public Health, in a soil mixture consisting of loamy soil, leaf mold, and pumice stone at a ratio of 6:3:1, in pots or planters. Unfortunately, Pa-7 and -9 died in July, 2011. In addition, *P. bracteatum* (Pa-13) and 2 types of *P. orientale* (Pa-14 and -15) plants at their flowering stage were used as reference species (Table 1; Fig. 1). Pa-13, -14, and -15 have been cultivated as stock plants at the Medicinal Plant Garden, Tokyo Metropolitan Institute of Public Health, while Pa-13 and -14 were kindly provided by the Tsukuba Division of the Research Center for Medicinal Plant Resources, and Pa-15 was obtained from the University of Oxford Botanic Garden as a seed.

**Table 1:** Plant materials used in the experiment

Plant number	Plant name at introduction	Collection time	Means of acquisition
Pa-1	Oriental poppy ‘Beauty of Livermere’	May 2011	Market in the Tokyo Metropolitan area
Pa-2	Oriental poppy ‘Beauty of Livermere’	May 2011	Market in the Tokyo Metropolitan area
Pa-3	Oriental poppy ‘Beauty of Livermere’	May 2011	Market in the Tokyo Metropolitan area
Pa-4	Oriental poppy ‘Beauty of Livermere’	May 2011	Market in the Tokyo Metropolitan area
Pa-5	Oriental poppy ‘Beauty of Livermere’	May 2011	Market in the Tokyo Metropolitan area
Pa-6	Oriental poppy ‘Beauty of Livermere’	May 2011	Market in the Tokyo Metropolitan area
Pa-7	Oriental poppy ‘Beauty of Livermere’	May 2011	Market in the Tokyo Metropolitan area
Pa-8	Oriental poppy ‘Beauty of Livermere’	May 2011	Market in the Tokyo Metropolitan area
Pa-9	Oriental poppy ‘Beauty of Livermere’	May 2011	Market in the Tokyo Metropolitan area
Pa-10	Oriental poppy ‘Beauty of Livermere’	May 2011	Market in the Tokyo Metropolitan area
Pa-11	Oriental poppy ‘Royal Wedding’	May 2011	Market in the Tokyo Metropolitan area
Pa-12	Oriental poppy ‘Prinzessin Victoria Louise’	May 2011	Market in the Tokyo Metropolitan area
Pa-13	*P. bracteatum*	April 2005	RCMPR^a^
Pa-14	*P. orientale*	August 2011	RCMPR^b^
Pa-15	*P. orientale*	May 2002	UOBG^c^

^a^Transferred from Tsukuba Division, Research Center for Medicinal Plant Resources, National Institute of Biomedical Innovation

^b^Transferred from Hokkaido Division, Research Center for Medicinal Plant Resources, National Institute of Biomedical Innovation

^c^Introduced by seed exchange from the University of Oxford Botanic Garden

### Morphological observation

For all of the plant materials (Pa-1 to -15), morphological characteristics were examined, including petal color, number of bract leaves, presence of bristles on sepals, and morphology of flower buds. Morphological observation was also made for bristles on the sepals and on the epidermis and for the stomata of the rosette leaves by using a scanning electron microscope (SEM) TM3000 (Hitachi High-Technologies Corp., Tokyo, Japan). The stomata length of each plant material was obtained as an average of values for 20 stomata.

Because Pa-3, -4, -5, -6, -8, -11, and -12 were at the vegetative stage when purchased, the morphological observation was repeated after reaching the flowering stage.

### Plastid subtype identity analysis

**Fig. 2:**
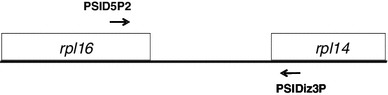
Primer pairs used for the determination of PSID5P2 and PSIDiz3P

The DNA sequence of plastid subtype identity (PS-ID) was analyzed for Pa-1, -3, -7, -10, -11, -12, -13, -14, and -15 according to the method of Hosokawa et al. [5] for the species of *Papaver*. Total DNA was extracted from fresh leaves by the modified cetyltrimethylammonium bromide (CTAB) method [6], and PS-ID sequences were amplified using primer pairs of PSID5P2: 5′-GTAGCCGTTGTTAAACCAGGTCGAATACTTTATGAAAT-3′, and PSIDiz3P: 5′-ACAGCAACAATAACGTCACCAATATGAGCATATCG-3′ (Fig. 2). Polymerase chain reaction (PCR) was performed using a thermal cycler TP2000 (Takara Bio Inc., Shiga, Japan) with the following temperature conditions; 32 cycles of 94 °C for 1 min, 51 °C for 1 min, and 72 °C for 2 min, followed by 72 °C for 10 min. Analysis of the sequences of the 9 plant materials examined was performed using Genetyx Mac genetic information analysis software version 15.0.5 (Genetyx Corp., Tokyo, Japan).

### Analysis of thebaine and isothebaine

Liquid chromatography–mass spectrometry (LC/MS) analysis was performed to identify thebaine and isothebaine in Pa-1 to -15.

As standard reagents, thebaine and isothebaine were purchased from Daiichi Sankyo Co., Ltd. (Tokyo, Japan) and from Apin Chemicals Ltd. (Oxfordshire, UK), respectively. Formic acid in acetonitrile (0.1 %, v/v, LC/MS grade) and all other chemicals (analytical grade) were purchased from Wako Pure Chemical Industries, Ltd (Osaka, Japan).

Sample solutions for the LC–MS analysis were prepared according to the methods of Ohnuki et al. [7]. A portion of each sample with an approximate weight of 200 mg was extracted with 8 mL of 75 % (v/v) ethanol and transferred to a centrifuge tube, where 1 mL of 27 % ammonium solution (aqueous) was added, and then the tube was shaken for 30 min followed by centrifugation at 3,000 rpm for 10 min. The supernatant was transferred to a 25-mL volumetric flask. The sediment in the centrifuge tube was re-extracted with 8 mL of 75 % ethanol. The supernatants were combined, and the final volume was adjusted to 25 mL with 75 % ethanol. After gentle shaking, the solution was passed through a 0.20-μm filter Millex LG (EMD Millipore Corp., MA, USA).

The LC–MS analysis was performed in an electrospray ionization (ESI) mode on an Acquity LC instrument connected to a quadrupole mass detector (Waters Corp., MA, USA). The test solution was analyzed using a Capcell Pak C18 IF2 column (length, 50 mm; internal diameter, 2.1 mm; particle size, 2.2 µm) (Shiseido Co., Ltd., Tokyo, Japan) at 40 °C. The LC gradient solutions were composed of a mobile phase A (5 mM ammonium formate buffer, pH 3.5, in water/acetonitrile, 95:5, v/v) and a mobile phase B (0.1 % formic acid in acetonitrile). With a flow rate was 0.6 mL/min, 100 % mobile phase A was initially run for 30 s, and then the final condition of a mixture of 10 % mobile phase A and 90 % mobile phase B was run for 30–411 s with a linear gradient setting. The injection volume was 1 µL. ESI mass analysis in positive mode was used for identification of the target compounds. Nitrogen gas supplied from a N_2_ generator was used for desolvation at 450 °C. The ion source temperature and the cone voltage were 150 °C and 20 V, respectively. The MS data were recorded with a range of mass-to-charge ratios (*m/z*) of 50–500 in the scan mode. Thebaine and isothebaine were detected at 1.96 and 1.83 min, respectively, and the minimum limit of detection (signal-to-noise ratio, *S*/*N* = 5) for each compound with the same ion [M+H]^+^ *m/z* 312 was 2 ng.

### Measurement of relative nuclear DNA content

The nuclear DNA content of Pa-1 to -15 was measured by FCM analysis using a CyFlow Type PA (Partec GmbH, Münster, Germany) according to the method of Galbraith et al. [8]. Leaf tissue of approximately 5 × 5 mm was chopped with a razor blade and mixed with 1.0 mL of a nuclear staining solution, consisting of 10 mM tris(hydroxymethyl)aminomethane (Tris), 50 mM trisodium citrate dehydrate, 2 mM MgCl_2_·6H_2_O, 1 % (w/v) polyvinylpyrrolidone k-30, 0.1 % (v/v) Triton X-100, and 2.5 mg/L 4′,6-diamidino-2-phenylindole dihydrochloride (DAPI), at pH 7.5 in a plastic Petri dish. The sample solution was then filtered through a 30-µm nylon mesh to remove cell debris. After incubating for 5 min, the stained nuclear suspensions were subjected to FCM analysis to determine relative nuclear DNA content. For each sample, at least 5,000 nuclei were counted, and the peak position was expressed as a value relative to that of *Trifolium repens* L., the internal standard.

## Results

### Morphological examination

The 3 *Papaver* species of section *Oxytona* are usually classified based on several morphological characteristics, such as the presence or absence of bract and petal color, as follows [2]:

1. *P. bracteatum*: the flower has 3–8 bracts and dark red petals. The bristles of the calyx are broad and triangular from the base, and non-erect.
2. *P. orientale*: the flower does not have bracts. The petals are usually unmarked, but occasionally have pale violet or white marks. The bud droops. Cauline leaves are not present on the upper third of a stem.
3. *P. pseudo*-*orientale*: The following 2 subtypes comprise this species.
  Subtype A: the flower has 1–4 bracts and orange to orange-red, “scarlet”, petals. The bristles of the calyx are slender and subpatent.
  Subtype B: the flower does not have bracts. The petals usually have broad rectangular black marks, but occasionally are unmarked. The buds are either erect or droop.
  Cauline leaves are present on the upper third of a stem.

The morphological characteristics and results of the identification of species for Pa-1 to -15 are summarized in Tables 2 and 3, respectively. Pa-1 to -10 and -13 had flowers with dark red petals and ~4–7 bracts. The bristles of the calyx were not erect, and the buds were erect. These morphological characteristics agreed well with those of *P. bracteatum*. In contrast, Pa-11, -12, and -15 had no bracts, and cauline leaves were present in the upper third of the stem. Pa-11 buds were erect and Pa-12 and -15 buds were not erect. These characteristics agreed well with those of *P. pseudo*-*orientale* (subtype B). Petal colors were white, pale orange, and deep orange in Pa-11, -12, and -15, respectively. In the case of Pa-15, it had originally been introduced into the Medicinal Plant Garden, Tokyo Metropolitan Institute of Public Health, as a seed of *P. orientale* The present study found that this was an incorrect identification, and we re-identified Pa-15 as *P. pseudo*-*orientale* (subtype B), mainly because of the position of the leaves on the stem. The morphological characteristics of Pa-14 were drooped buds, unmarked petals, and absence of cauline leaves on the upper third of a stem, which agreed well with that of *P. orientale*.

**Table 2:** Morphological characteristics of plant materials

Plant number	Color of petals	Number of bracts	Bristles of calyx	Buds	Color of marks of petals	Color of pollen	Number of petals
Pa-1^a^	Dark red	5	Non-erect	Erect	Dark purple	Dark purple	5
Pa-2^a^	Dark red	6	Non-erect	Erect	Dark purple	Dark purple	6
Pa-3^b^	Dark red	6	Non-erect	Erect	Dark purple	Dark purple	6
Pa-4^b^	Dark red	7	Non-erect	Erect	Dark purple	Dark purple	6
Pa-5^b^	Dark red	5	Non-erect	Erect	Dark purple	Dark purple	5
Pa-6^b^	Dark red	5	Non-erect	Erect	Dark purple	Dark purple	5
Pa-7^a^	Dark red	4	Non-erect	Erect	Dark purple	Dark purple	4
Pa-8^b^	Dark red	4	Non-erect	Erect	Dark purple	Dark purple	5
Pa-9^a^	Dark red	5	Non-erect	Erect	Dark purple	Dark purple	4
Pa-10^a^	Dark red	5	Non-erect	Erect	Dark purple	Dark purple	6
Pa-11^b^	White	0	Subpatent	Erect	Unmarked	Dark purple	6
Pa-12^b^	Pale orange	0	Subpatent	Droop	Black	Dark purple	6
Pa-13^a^	Dark red	5	Non-erect	Erect	Dark purple	Dark purple	5
Pa-14^b^	Pale orange	0	Subpatent	Droop	Unmarked	Gray	4
Pa-15^a^	Deep orange	0	Subpatent	Droop	Unmarked	Dark purple	4

^a^Confirmed flowering in May 2011

^b^Confirmed flowering in May 2012

**Table 3:** Species identification of plant materials by morphology, PS-ID, thebain and isothebaine content, and relative nuclear DNA content

Plant number	Morphology	PS-ID	Thebaine	Isothebaine	Relative nuclear DNA content
Pa-1	*P. bracteatum*	*P. bracteatum*	+	−	2.10
Pa-2	*P. bracteatum*	–	+	−	2.17
Pa-3	*P. bracteatum*	*P. bracteatum*	+	−	2.17
Pa-4	*P. bracteatum*	–	+	−	2.16
Pa-5	*P. bracteatum*	–	+	−	2.13
Pa-6	*P. bracteatum*	–	+	−	2.19
Pa-7	*P. bracteatum*	*P. bracteatum*	+	−	2.17
Pa-8	*P. bracteatum*	–	+	−	2.15
Pa-9	*P. bracteatum*	–	+	−	2.13
Pa-10	*P. bracteatum*	*P. bracteatum*	+	−	2.16
Pa-11	*P. pseudo*-*orientale*	*P. pseudo*-*orientale*	+	−	5.58
Pa-12	*P. pseudo*-*orientale*	*P. pseudo*-*orientale*	−	+	5.56
Pa-13	*P. bracteatum*	*P. bracteatum*	+	−	2.12
Pa-14	*P. orientale*	*P. orientale*	+	−	3.62
Pa-15	*P. pseudo*-*orientale*	*P. pseudo*-*orientale*	−	+	5.72

**Fig. 3:**
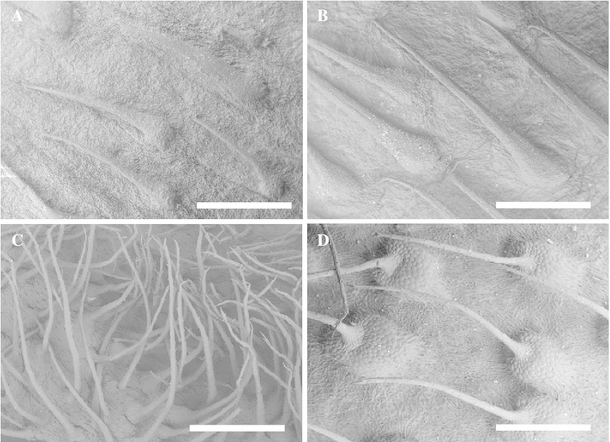
Representative SEM photomicrographs of the bristles of a calyx. **a** Pa-1; **b** Pa-13; **c** Pa-14; **d** Pa-15. *Bar* 1 mm

SEM revealed differences in the bristle morphology of the calyx (Fig. 3). Pa-1 to -10 and -13 had triangular bristles that were not erect, which agreed well with the typical morphology of *P. bracteatum* (Fig. 3a, b). In contrast, Pa-14 had thin and erect bristles (Fig. 3c), agreeing with the typical characteristics of *P. orientale*, whereas Pa-11, -12, and -15 had rather erect and slender bristles with swollen bases on the calyx (Fig. 3d), agreeing well with the characteristics of *P. pseudo*-*orientale* (subtype B).

**Fig. 4:**
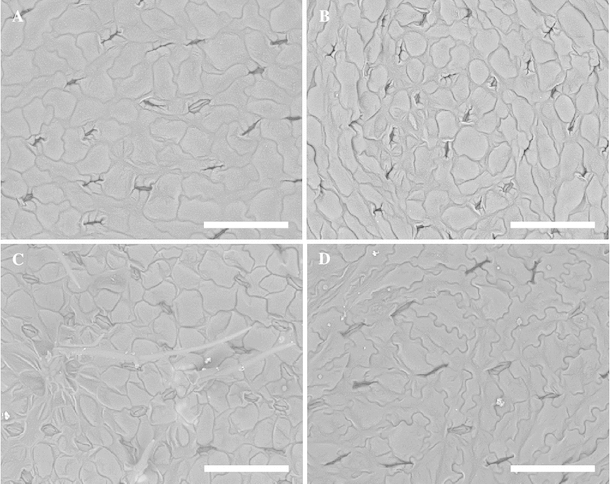
Representative SEM photomicrographs of the stomata. **a** Pa-1; **b** Pa-13; **c** Pa-14; **d** Pa-15. *Bar* 150 μm

Figure 4 shows the morphology of the leaf epidermis and stomata under SEM. According to Goldblatt, the stomata lengths of *P. bracteatum* are 20–32 µm, those of *P. orientale* are 32–45 µm, and those of *P. pseudo*-*orientale* are 43–60 µm [2]. In this study, the mean stomata length of Pa-13 was 28.7 µm, and those of Pa-1 to -10 averaged 31.8 µm. These results agree with Goldblatt’s data on *P. bracteatum*. The mean stomata length of Pa-14 was 34.8 µm, which agrees with Goldblatt’s reporting size of *P. orientale*. The mean stomata length of Pa-15 was 48.7 µm, which agrees with Goldblatt’s reporting size of *P. pseudo*-*orientale*. The mean stomata length of Pa-11 was 39.5 µm, and that of Pa-12 was 34.7 µm. These results agree with Goldblatt’s reporting size of *P. orientale*, and thus did not agree with their morphological identification outcomes.

### PS-ID analysis

**Fig. 5:**
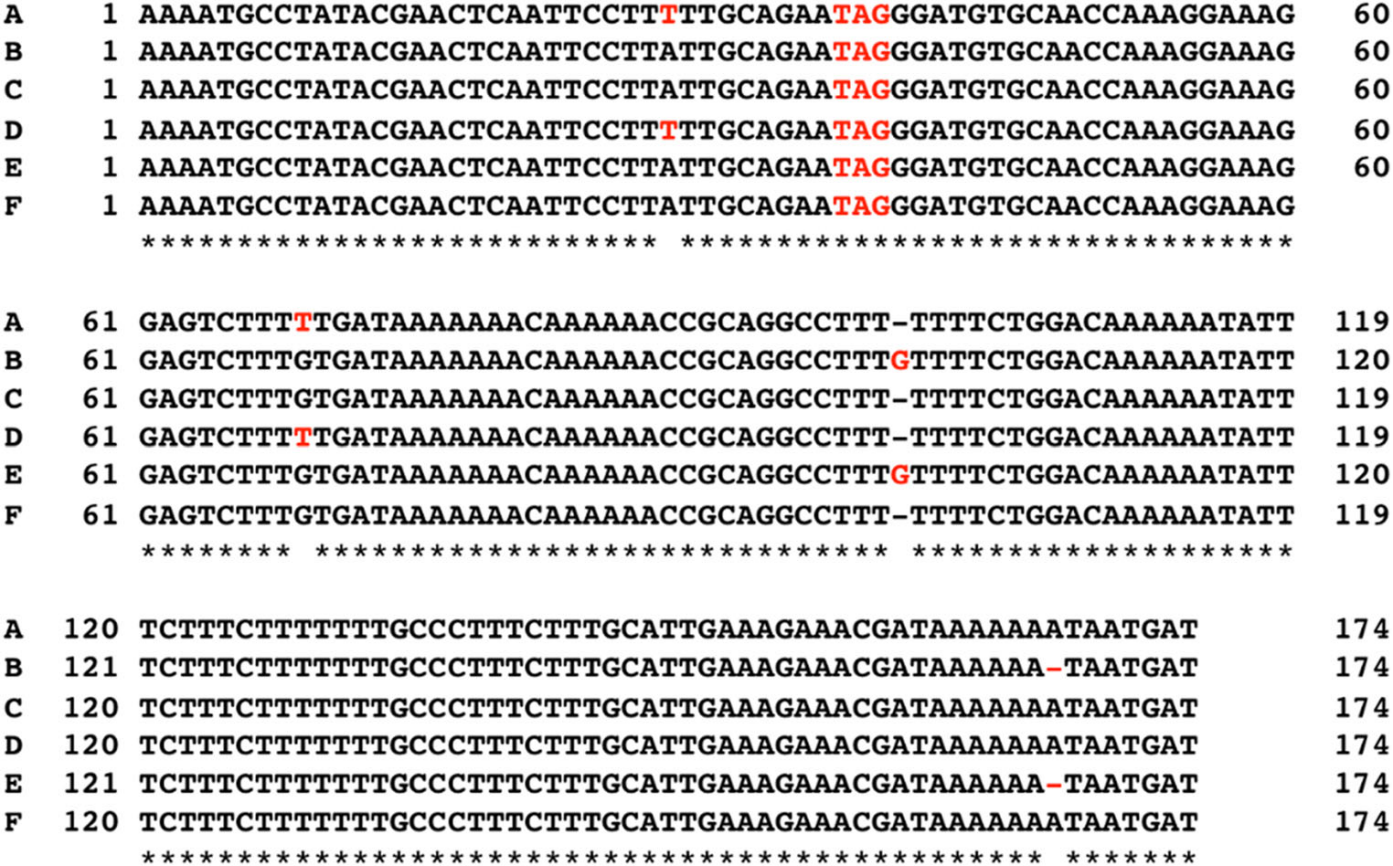
Alignments of PS-ID sequences for plants of section *Oxytona*. **a** *P. bracteatum*; **b** *P. orientale*; **c** *P. pseudo*-*orientale*; **d** Pa-13; **e** Pa-14; **f** Pa-15

The PS-ID sequences of 9 plant materials (Pa-1, -3, -7, -10, and -11 to -15) were compared with those previously reported for *P. bracteatum*, *P. orientale*, and *P. pseudo*-*orientale* by Hosokawa et al. [5] (Fig. 5). The PS-ID sequences of Pa-1, -3, -7, -10, and -13 agreed with that of *P. bracteatum*. Pa-14 had the same sequence as *P. orientale*. Pa-11, -12, and Pa-15 had sequences compatible with *P. pseudo*-*orientale*. These results agreed well with the findings obtained from the morphological identification (Table 3).

### Thebaine and isothebaine analyses

Table 3 shows the results of the analysis for the presence of thebaine and isothebaine in the leaves of Pa-1 to -15. According to Milo et al. [3] and Sariyar [9], the major alkaloid of *P. bracteatum* is thebaine, whereas those of *P. orientale* and *P. pseudo*-*orientale* are oripavine and isothebaine, respectively. In the present study, thebaine was detected in all plant materials except Pa-12 and -15, and the opposite was found for isothebaine which was only detected in Pa-12 and -15. Although Pa-11 was identified as *P. pseudo*-*orientale* based on the morphological characteristics and PS-ID analysis, thebaine, but not isothebaine, was detected. Likewise, Pa-14 was identified as *P. orientale* based on the morphological characteristics and PS-ID analysis, but thebaine was detected.

### FCM analysis

**Fig. 6:**
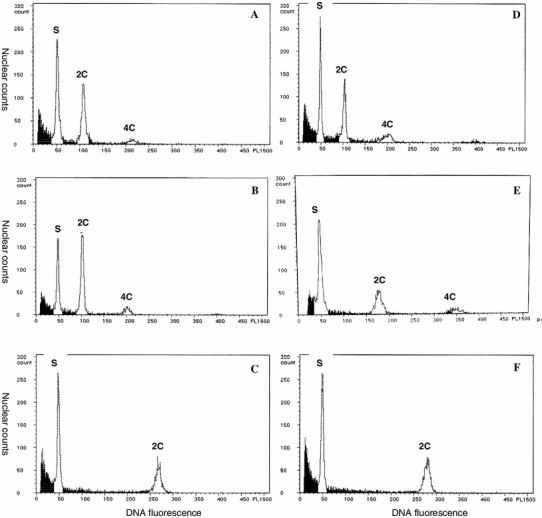
Flow cytometric profiles showing relative nuclear DNA content. **a** Pa-1; **b** Pa-5; **c** Pa-12; **d** Pa-13; **e** Pa-14; **f** Pa-15. *S* standard

The relative nuclear DNA contents of Pa-13, -14, and -15, introduced and presently confirmed or re-identified by the aforementioned analyses as *P. bracteatum*, *P. orientale,* and *P. pseudo*-*orientale*, were determined to be 2.12, 3.62, and 5.72, respectively (Table 3; Fig. 6d–f). On the other hand, the DNA contents of the plant materials collected from the market, Pa-1 to -10, fell into a narrow range of values (2.10–2.19), almost identical to the standard value of Pa-13, *P. bracteatum* (Table 3; Fig. 6a, b, d). In contrast, the DNA contents of Pa-11 and Pa-12 demonstrated close values (5.56–5.58), almost identical to the standard value of Pa-15, *P. pseudo*-*orientale* (Table 3; Fig. 6c, f). These results agreed well with the classification obtained from the morphological and PS-ID identification.

## Discussion

The present study performed a comparative identification of 12 commercially obtained plant materials (Pa-1 to -12) and 3 reference plants (Pa-13 to -15), all species of the genus *Papaver* section *Oxytona*, using a variety of classical and contemporary techniques. The morphological characteristics and analysis of PS-ID sequences of chloroplast DNA were in agreement and identified Pa-1 to -10 and -13 to be *P. bracteatum*, Pa-14 to be *P. orientale*, and Pa-11, -12, and -15 to be *P. pseudo*-*orientale*. Furthermore, a recently introduced technique, FCM analysis, revealed results identical to those obtained by the morphological and PS-ID analyses and thus clearly discriminated narcotic *P. bracteatum* from the other 2 species of section *Oxytona*. The most important and useful point for FCM analysis is that this method can be conducted using only leaves and thus is applicable to plants even at their vegetative stage, without flowers.

On the other hand, the present study showed that the species of 2 plants (Pa-11 and -12) could not be correctly identified when judged by their stomata lengths in SEM. It is thus indicated that the stomata length cannot be used as one of the main indicators for the identification of the species of the genus *Papaver* section *Oxytona*. Nevertheless, such a parameter may still be used as a subsidiary indicator.

A similar problem may be present regarding the thebaine and isothebaine analyses. Plant Pa-11 was identified as *P. pseudo*-*orientale* by good consensus of the morphological, PS-ID, and FCM analyses, but it contained thebaine, not isothebaine. Ohnuki et al. [7], however, recently reported that thebaine was detected in several individual plants sold under the name of oriental poppy. It is, therefore, possible that there may be a substrain of *P. pseudo*-*orientale* containing thebaine. Thus, this result suggests that the identification of *P. bracteatum* cannot be determined solely by the presence or absence of thebaine.

In conclusion, FCM analysis is a simple and useful method for quickly and clearly identifying the 3 species of the genus *Papaver* section *Oxytona*, using very small tissue samples. This method is applicable to living plants irrespective of their age, and can therefore detect narcotic species of *P. bracteatum* even at the immature stages with rosette leaves before flowering.
